# Supplementary material for: Wild worm embryogenesis harbors ubiquitous polygenic modifier variation
Source: eLife. 2015 Aug 22;4:e09178. doi: 10.7554/eLife.09178 (PMC4569889; doi:10.7554/eLife.09178)
Supplement: Supplementary file 2. — The LD column indicates clusters of SNPs in strong disequilibrium with each other (R2 > 0.90) across our test strains. A source data file has been deposited at Dryad under doi:10.5061/dryad.d5j06. DOI: http://dx.doi.org/10.7554/eLife.09178.012 [file elife-09178-supp2.docx]

**Supplementary file 2**

This extended table reports genome-wide SNPs associated with hatching phenotypes with

p-values < 0.0001 and < 1.64x10^-5^ (*). The LD column indicates clusters of SNPs in strong disequilibrium with each other (R^2^ > 0.90) across our test strains.

| LD | SNP | Silenced gene |
| --- | --- | --- |
| 1 | II_3364688 | *par-4* |
| 1 | II_3364737 | *par-4* |
| 1 | II_3376409 | *par-4* |
| 2 | II_4031280 | *cdc-37** |
| 2 | II_4031387 | *cdc-37** |
|  | II_4046789 | *cdc-37** |
| 3 | II_4130005 | *cdc-37* |
| 3 | II_4133328 | *cdc-37* |
| 4 | II_13213557 | *pkc-3* |
| 4 | II_13213572 | *pkc-3* |
| 5 | II_14211984 | *mel-28** |
| 5 | II_14212004 | *mel-28** |
| 5 | II_14281696 | *mel-28** |
|  | II_14447416 | *mel-28** |
|  | III_1546635 | *mel-28*, *pkc-3*, *rfc-3** |
| 6 | III_1557458 | *rfc-3** |
| 6 | III_1557477 | *rfc-3** |
| 6 | III_1571083 | *rfc-3** |
| 6 | III_1571372 | *rfc-3** |
| 7 | III_2781109 | *mel-28* |
|  | III_2864015 | *mel-28* |
| 7 | III_2916993 | *mel-28* |
| 7 | III_2952536 | *mel-28* |
| 7 | III_2994261 | *mel-28* |
| 7 | III_3051792 | *mel-28* |
|  | III_3083616 | *mel-28* |
| 7 | III_3380932 | *mel-28* |
| 8 | III_3484647 | *mel-28** |
| 9 | III_3498885 | *mel-28* |
| 9 | III_3501759 | *mel-28* |
|  | III_3575034 | *mel-28** |
| 8 | III_3578690 | *mel-28** |
| 8 | III_3587469 | *mel-28** |
|  | III_3618213 | *rpn-10* |
|  | III_3792289 | *mel-28* |
| 8 | III_3894285 | *mel-28** |
|  | III_4210576 | *mel-28** |
| 10 | III_4539993 | *mel-28** |
| 11 | III_4540978 | *mel-28** |
| 10 | III_4695574 | *mel-28** |
| 11 | III_4786311 | *mel-28** |
| 10 | III_4836084 | *mel-28** |
| 11 | III_5269824 | *mel-28** |
| 11 | III_10456811 | *mel-28** |
| 11 | III_10456812 | *mel-28** |
|  | IV_3981542 | *car-1* |
|  | IV_4062151 | *skn-1* |
| 12 | IV_4071456 | *car-1**, *mom-5*, *rpn-10*, *rpn-9**, *skn-1** |
|  | IV_4081026 | *car-1**, *rpn-9* |
| 12 | IV_4086041 | *car-1**, *mom-5*, *rpn-9**, *rpn-10*, *skn-1** |
| 13 | IV_4096423 | *car-1* |
| 13 | IV_4096424 | *car-1* |
| 14 | IV_4258798 | *par-6* |
|  | IV_4274791 | *rpn-10* |
| 14 | IV_4307316 | *par-6* |
| 14 | IV_4307503 | *par-6* |
| 14 | IV_4362684 | *par-6* |
| 15 | IV_5190341 | *car-1*, *mom-2* |
| 15 | IV_5216441 | *car-1*, *mom-2* |
| 15 | IV_5393585 | *car-1*, *mom-2* |
| 16 | IV_5581671 | *car-1** |
| 15 | IV_5615328 | *car-1*, *mom-2* |
| 16 | IV_5683718 | *car-1** |
| 16 | IV_5715800 | *car-1** |
| 16 | IV_5754103 | *car-1** |
| 16 | IV_5756507 | *car-1** |
| 17 | IV_6388961 | *car-1**, *mom-2* |
| 17 | IV_6664407 | *car-1**, *mom-2* |
|  | IV_6954170 | *car-1* |
| 18 | IV_6966989 | *car-1*, *mom-2** |
| 18 | IV_7147497 | *mom-2* |
| 18 | IV_7341696 | *mom-2* |
| 19 | IV_7548446 | *car-1** |
| 19 | IV_8074405 | *car-1** |
| 19 | IV_8089586 | *car-1** |
|  | IV_8249673 | *car-1*, *par-6** |
| 20 | IV_8263671 | *mom-2* |
| 20 | IV_8953909 | *mom-2* |
| 20 | IV_9473809 | *mom-2* |
| 20 | IV_9955132 | *mom-2* |
| 21 | IV_10211735 | *mom-2* |
| 20 | IV_10390698 | *mom-2* |
| 20 | IV_10547451 | *mom-2* |
| 21 | IV_10662438 | *mom-2* |
| 21 | IV_10671512 | *mom-2* |
| 20 | IV_10767228 | *mom-2* |
| 21 | IV_10783635 | *mom-2* |
| 21 | IV_10804503 | *mom-2* |
| 20 | IV_11077450 | *mom-2* |
| 21 | IV_11083410 | *mom-2* |
|  | IV_12996729 | *mom-2* |
|  | IV_13344021 | *mom-2* |
|  | V_16284380 | *skr-2* |
| 22 | V_16949076 | *lag-1* |
| 22 | V_16949111 | *lag-1* |
| 22 | V_16949115 | *lag-1* |
| 22 | V_16949121 | *lag-1* |
| 22 | V_16949123 | *lag-1* |
| 22 | V_16965822 | *lag-1* |
|  | V_18158643 | *par-2* |
| 23 | V_19918177 | *mel-28* |
| 23 | V_19920632 | *mel-28* |
| 23 | V_19920646 | *mel-28* |
| 23 | V_19920656 | *mel-28* |
| 23 | V_19920657 | *mel-28* |
| 23 | V_19925044 | *mel-28* |
| 23 | V_19925047 | *mel-28* |
|  | V_20191200 | *mel-28* |
| 23 | V_20391136 | *mel-28* |
| 24 | X_927222 | *pkc-3**, *rfc-3* |
| 24 | X_1016246 | *pkc-3**, *rfc-3* |
| 25 | X_1128868 | *rfc-3* |
| 25 | X_1129768 | *rfc-3* |
| 24 | X_1216610 | *pkc-3**, *rfc-3* |
| 24 | X_1218323 | *pkc-3**, *rfc-3* |
| 25 | X_1329775 | *rfc-3* |
| 24 | X_1411338 | *pkc-3**, *rfc-3* |
| 26 | X_9069608 | *par-4* |
| 26 | X_9088837 | *par-4* |
| 26 | X_9179389 | *par-4* |
| 26 | X_9273789 | *par-4* |
| 26 | X_9291768 | *par-4* |
| 26 | X_9350318 | *par-4* |
| 26 | X_9401674 | *par-4* |
| 26 | X_9497982 | *par-4* |
| 27 | X_15977592 | *par-6* |
| 27 | X_15977631 | *par-6* |
| 27 | X_17475626 | *par-6* |
| 27 | X_17478673 | *par-6* |
